# Heat therapy: Strategies to enhance thermal comfort without compromising benefits

**DOI:** 10.1113/EP093030

**Published:** 2025-07-22

**Authors:** Christof A. Leicht

**Affiliations:** ^1^ School of Sport, Exercise and Health Sciences Loughborough University Loughborough UK

Regular passive exposure to heat, using methods like sauna bathing or hot water immersion, has been linked to a reduced risk of cardiovascular disease (Laukkanen et al., 2015; Ukai et al., 2020). Whilst the epidemiological evidence is less robust than for physical activity interventions, both heat therapy and exercise hence represent non‐pharmacological tools with the capacity to benefit health. Especially older laboratory studies investigating the effects of passive exposure to heat often employed comparably high temperatures (resulting in a ≥1.5°C core temperature increase) and/or prolonged exposure times (≥60 min) (to cite two particularly taxing ones: Laing et al., 2008; Oehler et al., 2001). Such intense protocols can lead to changes in health‐related physiological outcomes. Acute increases in pro‐ and anti‐inflammatory cytokine concentration, blood flow and shear stress have been observed, alongside long‐term improvements in the resting inflammatory profile, glycaemic control, vascular function and blood pressure (Hoekstra et al., 2020; Pizzey et al., 2021). Subjective participant feedback regarding intervention tolerability is not available for most older investigations, and one might think that the idea of relaxing in a hot bath, rather than sweating it out in the gym, may be appealing to many. However, the experience of whole‐body passive heating can in fact be taxing and uncomfortable. Indeed, more recent investigations, assessing the associated thermal comfort for the historically often‐used ∼60‐min protocol to raise core temperature by ∼1–2°C, report participant feedback such as ‘it was intensely hot’, or worse – ‘too hot’, ‘too uncomfortable’ and ‘dreadful’ (Mansfield et al., 2021; Su, Hoekstra, Leicht, 2024).

This presents a challenge. Exercise and behaviour‐change research emphasises the importance of the subjective experience for long‐term adherence – an essential factor in achieving sustained health benefits (Ekkekakis et al., 2011). Optimising the subjective experience of passive heat exposure is therefore critical, yet has not often been the focus of heat therapy research.

A promising approach is the reduction of skin temperature, particularly in areas that are sensitive to heat. Methods like localised cooling using frozen cool packs (Hoekstra et al., 2021) or fans (Mansfield et al., 2021; Steward et al., 2023) have shown potential to improve thermal comfort. We have further shown that restricting cooling to the small, highly sensitive region of the face can enhance comfort whilst the effects on core body temperature are negligible, thereby maintaining the desired physiological responses (Su, O'Donnell, Hoekstra, et al., 2024). In contrast, cooling larger body areas can diminish the overall heat load, reduce blood flow to the chilled area, reduce local tissue temperature, and therefore reduce some physiological responses (Hoekstra et al., 2021) – a case in which better tolerability comes at the undesired cost of physiological effectiveness.

Local cooling during hyperthermic interventions may particularly benefit females. Females have a narrower range in which they feel thermally comfortable than males – feeling chillier sooner when temperatures drop and feeling hotter sooner when temperatures rise. Local cooling during passive heating can mitigate this sex difference in thermal perceptions, levelling the playing field for women who experience more discomfort than their male counterparts using traditional heat therapy approaches (Su, O'Donnell, Hoekstra, et al., 2024).

Skin temperature also plays a part in the perception of different heating methods. For instance, water temperature for hot water immersion is lower than sauna air temperature for the same increase in core temperature. As a result, skin temperature is higher for sauna use, and hot water immersion has been rated as more tolerable in a direct modality comparison (Su, Hoekstra, Leicht 2024). Whilst personal preference likely also plays a part, such findings may help nudge people with no previous heat therapy experience to modalities and interventions that maximise comfort and adherence.

Returning to ‘real‐world’ observations, Laukkanen et al. (2015) observed an average sauna bathing duration of just under 15 min in their cohort of 2315 Finnish men. Whilst they report the biggest reductions in mortality in those bathing >19 min per session, this is far below the 60 min often investigated in laboratory studies, implying that in real life, shorter stimuli are used, which still confer health benefits.

In conclusion, laboratory passive heating study protocols using intense stimuli can result in health benefits, but may not necessarily work in the ‘real world’ as negative perceptions would likely reduce adherence. Whilst an adequate heat stimulus is necessary for beneficial adaptations, strategies to limit the skin temperature rise – especially in sensitive areas – can improve comfort. This, in turn, may result in better long‐term adherence and ultimately enhance the health benefits of heat therapy.

## AUTHOR CONTRIBUTIONS

Sole author.

## CONFLICT OF INTEREST

None declared.

## FUNDING INFORMATION

No funding was received for this work.
